# Is There an Effect of Methyl Donor Nutrient Supplementation on Metabolic Syndrome in Humans?

**DOI:** 10.3390/medsci8010002

**Published:** 2020-01-06

**Authors:** Naser A. Alsharairi

**Affiliations:** Menzies Health Institute Queensland, Heart, Mind & Body Research Group, Griffith University, P.O. Box 4222, Gold Coast 4222, Australia; naser.alsharairi@gmail.com

Metabolic syndrome (MetS), also termed insulin resistance syndrome, has been defined by World Health Organization (WHO) as a multi-factorial disorder characterized by a wide array of cardiometabolic risk factors that increase the risk of coronary heart diseases, diabetes mellitus, and stroke [1,2]. The most common risk factors of MetS include dyslipidemia, abdominal obesity, insulin resistance, high systolic blood pressure, high triglyceride levels, impaired fasting glycaemia, low plasma adiponectin levels, and low high-density lipoprotein (HDL). However, it remains debatable whether these factors constitute a particular syndrome or are surrogate markers for MetS clustering in individuals at particular risk [1].

A key aspect of MetS is its rising prevalence in the world’s children and adults, and the future health burden it places [1,2]. The high prevalence of MetS coincides with the upswing in obesity in developed and developing countries [2,3]. Although the cause of MetS is not well understood, it is thought that insulin resistance, dysregulate expression of insulin receptor substrate (IRS) genes, pancreatic β-Cell dysfunction, oxidative stress and glucose toxicity, chronic inflammation, gut microbiota, a high fat diet, and epigenetics may play a critical role in the etiology of MetS [4].

Epigenetics is a broad area of genetics that focuses on a phenomenon of heritable alterations in gene phenotype or expression. These epigenetic alterations do not require changes in the nucleotide sequence of DNA but rather involve epigenetic mechanisms such as posttranslational modifications of histone proteins and DNA methylation [5]. Together, these epigenetic modifications influence chromatin structure which plays a regulatory role of gene expression in a number of human diseases such as atherosclerosis, hypertension, inflammation [5,6], obesity, and diabetes [5,6,7].

DNA methylation relies on the cellular availability of methyl groups derived from diet in the one-carbon metabolic (OCM) pathway, by which methyltetrahydrofolate (methyl-THF), methionine, choline/betaine, and vitamins B12, B2, and B6 are closely interconnected to form methionine from homocysteine. The OCM pathway is driven by a number of DNA methyltransferase enzymes using S-adenosylmethionine (SAM) produced from methionine as a cofactor to transfer methyl groups to cytosine residue in DNA, leading to the formation of S-adenosylhomocysteine (SAH) [8,9].

Gene susceptibility to MetS in humans interacts with environmental factors, such as diet, which can induce changes in gene expression. Data from genetic epidemiological studies demonstrate that diet can modulate human genes and lead to an increased risk of developing MetS through association with genes responsible for inflammation, lipid-related markers, and obesity [10]. A considerable increase in the intake of methyl donor compounds that are due to niacin fortification, high meat consumption, and food additives may play a role in the development of MetS and its related diseases. The increased intake of these compounds may also be a significant risk factor for hyperhomocysteinemia, caused primarily by a deficiency of folate, which is an important component in DNA methylation. Oxidative stress and aberrant methylation status are considered as underlying pathogenic mechanisms of MetS, but how they exist has not been well discussed [11].

The pathogenesis of MetS involves both primary and secondary factors. The key pathogenic factors of MetS include systemic oxidative stress and dietary methyl consumer-induced methyl depletion. On the other hand, high fat and alcohol intake, stressors, decreased skin function, and low vegetable intake have been considered secondary factors. All of these factors result from methyl supply-intake imbalance and oxidative injury [11]. As a result, genetic alterations caused by diet or other factors may be a target for inducing novel therapeutic strategies for modulating the progression to MetS using dietary manipulation such as supplementation with methyl-donor nutrients. This raises the question of whether these supplements could have a beneficial effect on MetS and its components in humans. 

A large number of randomized control trials (RCTs) have focused on the effect of folate supplementation on MetS components in humans. They have mostly targeted adults. However, RCTs examining the effect of other supplements are limited. A systematic review and meta-analysis of 13 RCTs reported no significant effect for folate supplementation over a placebo on the lipid profile (triglycerides, total cholesterol, and systolic and diastolic blood pressure) in patients with metabolic diseases [12]. A recent systematic review and meta-analysis of 29 RCTs showed that folate supplementation alone compared to placebo (folate combination with other B vitamins) reduced the homeostasis model assessment for insulin resistance (HOMA-IR) and fasting insulin, but no effect was observed on glycated hemoglobin (HbA1c) and fasting glucose. The pool effect reported a positive correlation between absolute decreases in homocysteine concentrations and absolute decreases in HbA1c and fasting glucose concentrations after folate supplementation [13].

The most recent Cochrane review of 15 RCTs examined whether homocysteine-lowering interventions lowered the risk of cardiovascular diseases (CVD) among 71,422 adults with e.g., hypertension, low levels of high density lipoprotein (HDL) or high total cholesterol, and without established CVD. The study showed no effect of interventions from testing vitamins B9/folic acid, B6, or B12 supplements alone or in combination compared with placebo for preventing CVD [14]. A randomized double-blinded, placebo-controlled trial reported reduction in fasting glucose levels, insulin response to oral glucose, and intrahepatic triglyceride levels among adults with obesity and prediabetes (21–70 years) after supplementation with betaine (3300 mg twice daily for 10 days, followed 4950 mg twice daily for 12 weeks) compared to a placebo. However, no effect was observed on HbA1c and insulin sensitivity was not improved after betaine treatment compared to a placebo [15].

In conclusion, supplementation with folate might be beneficial for reducing insulin resistance and fasting insulin, but folate alone or in combination with vitamins B6 and B12 supplements had no effect on HbA1c, fasting glucose, total cholesterol, triglycerides, low HDL, and systolic and diastolic blood pressure. A low plasma homocysteine concentration has been associated with reduced HbA1c and fasting glucose concentrations after folate supplementation. Betaine supplementation resulted in lowering triglyceride and fasting glucose levels, but had no effect on insulin sensitivity and HbA1c.

Further RCTs regarding the effects of single or multi-methyl donor nutrient supplementation on MetS components in children and adults are needed. Gaining a better understanding of epigenetic regulation of gene expression in MetS would be useful to guide future RCTs for nutritional interventions based on methyl-donor supplements that may alter the epigenetic marks of genes, thereby modulating the progression of MetS.

## References

[1] Kassi E., Pervanidou P., Kaltsas G., Chrousos G. (2011). Metabolic syndrome: Definitions and controversies. BMC Med..

[2] Saklayen M.G. (2018). The global epidemic of the metabolic syndrome. Curr. Hypertens. Rep..

[3] DeBoer M.D. (2019). Assessing and managing the metabolic syndrome in children and adolescents. Nutrients.

[4] Xu H., Li X., Adams H., Kubena K., Guo S. (2018). Etiology of metabolic syndrome and dietary intervention. Int. J. Mol. Sci..

[5] Handy D.E., Castro R., Loscalzo J. (2011). Epigenetic modifications: Basic mechanisms and role in cardiovascular disease. Circulation.

[6] Muka T., Koromani F., Portilla E., O’Connor A., Bramer W.M., Troup J., Chowdhury R., Dehghan A., Franco O.H. (2016). The role of epigenetic modifications in cardiovascular disease: A systematic review. Int. J. Cardiol..

[7] Van Dijk S.J., Tellam R.L., Morrison J.L., Muhlhausler B.S., Molloy P.L. (2015). Recent developments on the role of epigenetics in obesity and metabolic disease. Clin. Epigenet..

[8] Niculescu M.D., Zeisel S.H. (2002). Diet, methyl donors and DNA methylation: Interactions between dietary folate, methionine and choline. J. Nutr..

[9] Anderson O.S., Sant K.E., Dolinoy D.C. (2012). Nutrition and epigenetics: An interplay of dietary methyl donors, one-carbon metabolism and DNA methylation. J. Nutr. Biochem..

[10] Ordovas J.M., Shen J. (2008). Gene-environment interactions and susceptibility to metabolic syndrome and other chronic diseases. J. Periodontol..

[11] Zhou S.S., Zhou Y.M., Li D., Lun Y.Z. (2011). Dietary methyl-consuming compounds and metabolic syndrome. Hypertens. Res..

[12] Tabrizi R., Lankarani K.B., Akbari M., Naghibzadeh-Tahami A., Alizadeh H., Honarvar B., Sharifi N., Mazoochi M., Ostadmohammadi V., Fatholahpour A. (2018). The effects of folate supplementation on lipid profiles among patients with metabolic diseases: A systematic review and meta-analysis of randomized controlled trials. Diabetes Metab. Syndr..

[13] Lind M.V., Lauritzen L., Kristensen M., Ross A.B., Eriksen J.N. (2019). Effect of folate supplementation on insulin sensitivity and type 2 diabetes: A meta-analysis of randomized controlled trials. Am. J. Clin. Nutr..

[14] Martí-Carvajal A.J., Solà I., Lathyris D., Dayer M. (2017). Homocysteine-lowering interventions for preventing cardiovascular events. Cochrane Database Syst. Rev..

[15] Grizales A.M., Patti M.E., Lin A.P., Beckman J.A., Sahni V.A., Cloutier E., Fowler K.M., Dreyfuss J.M., Pan H., Kozuka C. (2018). Metabolic effects of betaine: A randomized clinical trial of betaine supplementation in prediabetes. J. Clin. Endocrinol. Metab..

